# Black ginseng: a novel medicine for treating heart failure

**DOI:** 10.3389/fphar.2024.1429214

**Published:** 2024-07-18

**Authors:** Peiyuan Dou, Linlin Liu, Mozhu Jin, Jing Huang, Rose Makhotso Lekhooa, Xiaoku Ran, Xiaohui Yan

**Affiliations:** ^1^ State Key Laboratory of Component-based Chinese Medicine, Tianjin University of Traditional Chinese Medicine, Tianjin, China; ^2^ College of Pharmacy, Liaoning University of Traditional Chinese Medicine, Dalian, China; ^3^ Preclinical Drug Development Platform, Faculty of Health Sciences, North-West University, Potchefstroom, South Africa

**Keywords:** black ginseng and its fractions, heart failure, steroids, UPLC-QqQ-MS/MS, SCFAs

## Abstract

**Introduction:** Black ginseng (BG) was processed by “steaming and drying” (generally nine times) repeatedly to produce “rare saponins” and secondary ginsenosides. Both ginseng (GS) and red ginseng (RG) were commonly used in treating heart failure (HF), and the latter was confirmed to be more potent, implying the presence of rare ginsenosides that contribute positively to the treatment of heart failure. Previous research indicated that rare ginsenosides are more abundant in BG than in RG. Consequently, this study aims to investigate the effects of BG and its components on HF to elucidate the active substances and their underlying mechanisms in the treatment of HF.

**Methods:** The effects of BG and its fractions (water-eluted fraction (WEF), total saponin fraction (TSF), and alcohol-eluted fraction (AEF)) on rats with isoproterenol (ISO)-induced HF were explored, and steroids belonging to the hypothalamic–pituitary–adrenal (HPA) and hypothalamic–pituitary–gonadal (HPG) axes were determined quantitatively using the ultra-performance liquid chromatography–triple quadrupole tandem mass spectrometry (UPLC-QqQ-MS/MS) method. In addition, 16S rDNA sequencing was performed on the gut microbiota, followed by GC–MS analysis of short-chain fatty acids (SCFAs), and the biochemical indexes related to energy metabolism and the serum cyclic nucleotide system were also analyzed by ELISA.

**Results:** Based on a thorough evaluation of energy metabolism and the endocrine system, it was observed that the effects of BG components on the hypothalamic–pituitary–thyroid (HPT) and HPA axes were more pronounced. Notably, the treatment efficacy of the low dose of the total saponin fraction (TSFL), water decoction (WD), and high dose of the polysaccharide fraction (PSFH) was superior based on pharmacodynamic indicators such as brain natriuretic peptide (BNP), creatine kinase (CK), and estradiol (E2)/T). Furthermore, the WD and BG components exhibited significant effects on androgens (T and androstenedione (A4)). The TSFL group exerts an anti-inflammatory effect by regulating *Lactobacillus*/*Erysipelotrichales*. The WD, PSFH, and TSFL may impact inflammatory cytokines through the gut microbiota (*Lactobacillus*/*Erysipelotrichales*) and their metabolites (acetate and butyrate), exerting an anti-inflammatory effect.

**Discussion:** The BG and all its split components demonstrated varying levels of efficacy in alleviating HF, and TSF and PSF exhibited a significant protective effect on HF. The main active components in TSF were revealed to be ginsenosides Rk1, Rk3, 20-(S)-Rg3, and 20-(S)-Rh2 by the H9C2 cell experiment. The decoction of BG and its components exhibited a potent impact on androgen hormones, with an elevation trend. This phenomenon may be attributed to the activation of the eNOS-NO pathway through androgen regulation, thereby contributing to its anti-HF activities. The WD, PSFH, and TSFL may exert anti-inflammatory effects through the intestinal flora (*Lactobacillaceae*/*Erysipelotrichaceae*) and its metabolites (acetic acid and butyric acid), which affect the inflammatory factors. The different mechanisms of action of each component of HF also reflect the significance and necessity of the overall role of traditional Chinese medicine (TCM). Our research was the first to report that the E2/T is related to HF and can be used as an indicator to evaluate HF.

## 1 Introduction

The roots and rhizomes of *Panax ginseng* C. A. Mey., commonly known as ginseng (GS), are world-famous medicines. Three types of processed ginseng are commonly used in traditional Chinese medicine (TCM), *i.e.*, ginseng, sun-dried ginseng, and red ginseng (RG). More recently, black ginseng (BG) has been produced through the procedure of multiple repetitions of “steaming and drying” (generally nine times) fresh ginseng to produce “rare saponins” or secondary ginsenosides that are lower in red ginseng (red ginseng is the product of a one-time “steaming and drying” process applied to fresh ginseng). Previously, the comparison of the chemical changes in ginseng, red ginseng, and black ginseng and the examination of the medicinal nature and anticancer constituents in black ginseng (Zhu et al., 2019; Wan et al., 2022) indicated that the ginsenosides in red ginseng were partially transformed into rare or secondary ginsenosides, while they were almost completely transformed in black ginseng, with stronger anticancer activity as reported.

The traditional effect of GS and RG is to “reinvigorate the pulse for relieving *qi* depletion.” They are closely linked to anti-heart failure (HF) properties, and HF is a clinical manifestation of the deficiency of “heart-*qi*.” Both ginseng and red ginseng are commonly used in heart failure, with the latter confirmed to be stronger than the former (Peng et al., 2021). This tells us that rare ginsenosides should have a greater contribution to the treatment of heart failure, as rare ginsenosides are mostly responsible for their chemical differences.

Currently, BG has been produced industrially on a large scale in China and is used in accordance with the functions of ginseng. Thus, the examination of the anti-HF mechanism of action of BG and its substance basis is imperative to direct its clinical application and provide a basis for the development of new products. Research revealed that the hypothalamic–pituitary–adrenal (HPA) axis is involved in various actions of ginseng that relate to heart vascular protection through the modulation of glucocorticoid hormones (Li et al., 2014). Recently, research from our laboratory indicated that the medicinal nature (drug Xing) of ginseng, a unique index for measuring the efficacy of Chinese medicine, is associated with the HPA and hypothalamic–pituitary–thyroid (HPT) axes and energy metabolism. The nature of black ginseng was described as mild and warm, further revealing that the efficacy of ginseng is associated with the regulation of the HPA and HPT axes (Liu et al., 2024). Other groups have shown that the vascular protective action of ginseng is related to the gonadal receptors and testosterone (Zhou et al., 2011; Tostes et al., 2016), i.e., the hypothalamic–pituitary–gonadal (HPG) axis. Thus, the regulation of the HPT, HPA, and HPG axes has been assayed by endocrinological steroid hormones, which may be one of the mechanisms of action of ginseng on HF; however, how individual androgens and estrogens change after taking ginseng is still unclear in HF status. Recent research indicated that the “heart–gut axis” also participated in HF recovery (Liu J. et al., 2022), and the variations in the intestinal microflora diversity were generally assayed by 16S rDNA sequencing and short-chain fatty acids (SCFAs). To have better utilization of BG, this paper focuses on its mechanism and substance basis on HF induced by isoproterenol from the viewpoints of endocrinological hormones to reflect its effects on the HPA, HPT, and HPG axes; heart energy metabolism; and the intestinal microflora. It is for the first time that the mechanism of action of HF in black ginseng and its substance basis have been examined systematically.

## 2 Materials and methods

### 2.1 Chemicals

The ISO batch no. H2124300 is from Aladdin (Shanghai, China). The ELISA kits, including triiodothyronine (T3), thyroxine (T4), cyclic adenosine monophosphate (cAMP), cyclic guanosine monophosphate (cGMP), brain natriuretic peptide (BNP), creatine kinase (CK), interleukin-1(IL-1), adrenergic (A), estradiol (E2), testosterone (T), and ultrafine Na^+^–K^+^-ATPase detection kit (Batch No. 20230517) were purchased from the Nanjing Jian Cheng Institute of Biotechnology (Nanjing, China). The calibrators, including androstenedione (A4), 17-hydroxyprogesterone (17-OH-P), 17-hydroxypregnenolone (17-OH-PR), progesterone (P), pregnenolone, dehydroepiandrosterone (DHEA), aldosterone (ALDO), cortisol (COR), corticosterone (CORT), cortisone, and deoxycorticosterone (DOC), were acquired from Merck (Billerica, MA, United States), Cambridge Isotope Laboratories (Cambridge, MA, United States), and J&K (TRC, Toronto, Canada). The internal standards, including A4-13C3, 17-OH-P-d8, 17-OH-PR-13C2-d2, P-d9, pregnenolone-d4, DHEA-d6, ALDO-d8, COR-d4, CORT-d8, and 21-OH-P-d7, were also acquired from the same sources.

### 2.2 Plant materials and extraction methods

BG was provided by a local company (Zhongshutang Black Ginseng Company, Xifeng County, Liaoning Province, China). BG (310.5 g) was ground into powder and extracted twice with 3.1 L of water, with 2 hours for each cycle. After that, the water decoction (WD) was evaporated to 1,870.5 mL, and 467 mL of water decoction was separated as the WD group. Then, the rest of the water decoction was concentrated to 1,000 mL, and 95% ethanol (2.8 L) was added to the rest of the water decoction to separate the polysaccharide fraction. The polysaccharide fractions (PSFs) were filtered by suction and dried using a vacuum drying machine to obtain pure polysaccharides. The remaining water decoction part was subjected to macroporous adsorption resin D101 and eluted with 20% ethanol, 70% ethanol, and 95% ethanol. The 20% ethanol eluates were collected as the resin water-eluted fraction (WEF). The 70% ethanol eluates were collected as the total saponin fraction (TSF). The 95% ethanol eluates were collected as an alcohol-eluted fraction (AEF). All fractions were heated, lyophilized, and stored at −20°C. Furthermore, HPLC was used to analyze the main compounds of the fractions, and the results are shown in Supplementary Figures S1–S4, revealing the main constituents in each fraction.

### 2.3 Animals and experiment protocols

The Sprague–Dawley (SD) rats (male, 180–200 g) were provided by Changsheng Bio-Technique Co., Ltd., qualification number SCXK 2020-0001 (Benxi, 143 Liaoning, China). The temperature of the feeding room was kept at 20°C–25°C, and the relative humidity was 50%–65%. The rats were housed in cages and had free access to water and food. After 7 days of the observation period, the rats were divided into 12 groups (*n* = 11), including the control (CON) group, model (MOD) group, water decoction (WD) group (3.32 g/kg), low dose of the alcohol-eluted fraction (AEFL) group (0.22 × 10^−2^ g/kg), high dose of the alcohol-eluted fraction (AEFH) group (0.43 × 10^−2^ g/kg), low dose of the polysaccharide fraction (PSFL) group (46.78 × 10^−2^ g/kg), high dose of the polysaccharide fraction (PSFH) group (93.56 × 10^−2^ g/kg), low dose of the resin water-eluted fraction (WEFL) group (33.19 × 10^−2^ g/kg), high dose of the resin water-eluted fraction (WEFH) group (66.37 × 10^−2^ g/kg), low dose of the total saponin fraction (TSFL) group (3.13 × 10^−2^ g/kg), high dose of the total saponin fraction (TSFH) group (6.25 × 10^−2^ g/kg), and captopril (POS) group (9.22 mg/kg). The rats in the CON group were subcutaneously administered an equal volume of normal saline, while the rats in the other group were injected subcutaneously with ISO at a dose of 50 mg/kg/d for seven consecutive days (Liu X. et al., 2022). The split fraction-treated groups were administered for 14 days with the corresponding group assignment, and the specific concentration is shown in Table 1. The protocol was approved by the Liaoning University of Traditional Chinese Medicine’s Animal Ethics Committee, which approved all animal studies (approval no. 2020067).

**TABLE 1:** Concentration of the split fractions.

Sub-fraction	Yield (%)	Dose (mg/mL)
WEFL	19.99	33.18
WEFH		66.36
TSFL	1.88	3.13
TSFH		6.25
AEFL	0.13	0.22
AEFH		0.43
PSFL	28.21	470.00
PSFH		940.00

### 2.4 Weight-loaded swimming test

The animals were forced to swim in a tank until exhaustion on the 13th day after being treated with BG and its fractions. The endurance time from start to exhaustion was recorded.

### 2.5 Echocardiography

On the 21st day (administered for 14 days with BG and its fractions), echocardiography was conducted using the Vevo 1100 ultrasound device (VisualSonics, Canada). In brief, short-axis M-mode echocardiography was performed to measure parameters such as the left ventricular internal diameter in systole and diastole (LVID s and LVID d), left ventricular posterior wall thickness in systole and diastole (LVPW s and LVPW d), ejection fraction (EF), fractional shortening (FS), heart rate (HR), stroke volume, and cardiac output. Finally, the rats were euthanized. All images were analyzed using the Vevo 1100 Protocol-Based measurement software.

### 2.6 Histological analysis

Fresh myocardial tissue was fixed in 10% formalin and maintained in a buffer solution. After 72 h of dehydration in a gradient ethanol solution, the tissue was embedded in paraffin. The tissue block was then cut to a thickness of 4 μm. Myocyte hypertrophy was analyzed through hematoxylin–eosin (HE) staining, and the sizes of cardiomyocytes from each rat were detected at ×20 magnification using light microscopy. Collagen volume fraction was calculated as the percentage of collagen fiber area in the myocardial tissue using Masson’s trichrome staining, and finally, images were analyzed using Aipathwell (Wuhan Servicebio Technology CO., LTD.), from which the collagen volume fraction (CVF) of the myocardial tissue was calculated based on the following formula: CVF = collagen area/total observed area × 100%.

### 2.7 UPLC-QqQ-MS/MS analysis

A Waters^®^ Xevo™ TQS MS ACQUITY UPLC^®^ System was used to separate and measure each analyte using an ACQUITY UPLC BEH C8 Column, 100 mm × 2.1 mm, 1.7 μm (Milford Waters, MA, United States), which was coupled with a solid-phase extraction (SPE) unit. The SPE unit used an OASIS PRiME HLB μElution Column Plate (Milford Waters, MA, United States) and a 96-well 350 μL ACQUITY Collection Plate (Milford Waters, MA, United States). The filtrate was injected after filtration, and the autosampler temperature was maintained at 10°C. The mobile phase consisted of 0.3 mM NH_4_F (A) and methanol (B), and the elution gradient is shown in Table 2 (Wang et al., 2020). The steroid profile was based on electrospray ionization–mass spectrometry (ESI-MS) data combined with tandem mass spectrometry analysis in the positive or negative ion mode. Quantitative analysis was performed using multiple response monitoring (MRM) modes for nine analytes in the positive ion mode, namely, A4, 17-OH-P, P, pregnenolone, DHEA, COR, CORT, cortisone, and 21-OH-P, and for the two analytes ALDO and 17-OH-PR.

**TABLE 2:** Elution conditions for LC separation.

Time (min)	Flow (mL/min)	Phase A (%)	Phase B (%)
0	0.25	50	50
0.5	0.25	50	50
3	0.25	45	55
5.5	0.3	5	95
6.5	0.25	5	95
8	0.25	50	50

### 2.8 ELISA analysis

After collecting blood from the abdominal aorta, it was left to stand at room temperature for 2 h and then centrifuged at 3,000 rpm and 4°C for 15 min. The supernatant was then taken for analysis. The levels of BNP, CK, and IL-1 were measured to further evaluate and validate the anti-HF function of BG and its fractions. The levels of T3 and T4 were also measured, and their impact on the thyroid hormones was evaluated. The activity of AChE was measured to evaluate its effect on the nervous system, while the content of cAMP and cGMP was evaluated for their impact on the cyclic nucleotide system. The levels of E2, T, and A were also determined according to the manufacturer’s instructions. Finally, the heart Na^+^–K^+^-ATPase activity was determined according to the instructions of the assay kit, and the tissue protein content was determined using the Coomassie brilliant blue method.

### 2.9 Intestinal flora and SCFA analysis

The sequencing and analysis of intestinal flora are detailed in Supplementary Material S2. The short fatty acids in rat feces were determined according to Pang et al. (2021). The chromatographic column used was an Agilent HP-INNOWax Capillary Column (30 m × 0.25 mm, 0.25 μm). The injection volume was 1 μL, and the carrier gas was helium, which flowed at 1.0 mL/min. The electron bombardment ion source (EI) had an electron energy of 70 eV, a solvent delay of 3 min, and scanning modes of SCAN and SIM.

### 2.10 Cell viability assay

An embryonic rat cardiomyocyte line, H9C2, was purchased from the Chinese Academy of Sciences (Beijing). All cell lines were cultured at 37°C in 5% CO_2_ in humidified incubators in DMEM supplemented with 10% fetal calf serum. We obtained the following compounds from BG (Zhao et al., 2024): F4, Rk3, Rh4, 20-(S)-Rg3, 20-(S)-Rg3, Rk1, Rg5, 20-(S)-Rh2, 20-(R)-Rh2, and so on (12). The H9C2 cells were treated with the test compounds at concentrations of 0.1, 1, 10, and 50 μmol/L. The control group cells were cultured normally without any treatment. After 24 h of pretreatment, ISO treatment was performed in the drug group, while the blank group contained only the medium without cells. Cells were cultured in the conventional incubator for 48 h, and then 10 L of the CCK-8 solution was added to each well; then they were cultured at 37°C in 5% CO_2_ for 2 h. The OD was measured at 450 nm, and the cell activity was calculated.

### 2.11 PCA

Principal component analysis (PCA) was used to group and cluster all the *in vivo* data measured by ELISA. The results are visualized in the form of a score chart, where each point represents a separate sample.

### 2.12 Data analysis

In this study, all data are presented as means ±standard deviations (S.D.). One-way analyses of variance were performed using SPSS 25. The data were plotted using GraphPad Prism 8 (version 8.02).

## 3 Results

### 3.1 Effects of BG and its fractions on weight-loaded swimming time

**FIGURE 1:**
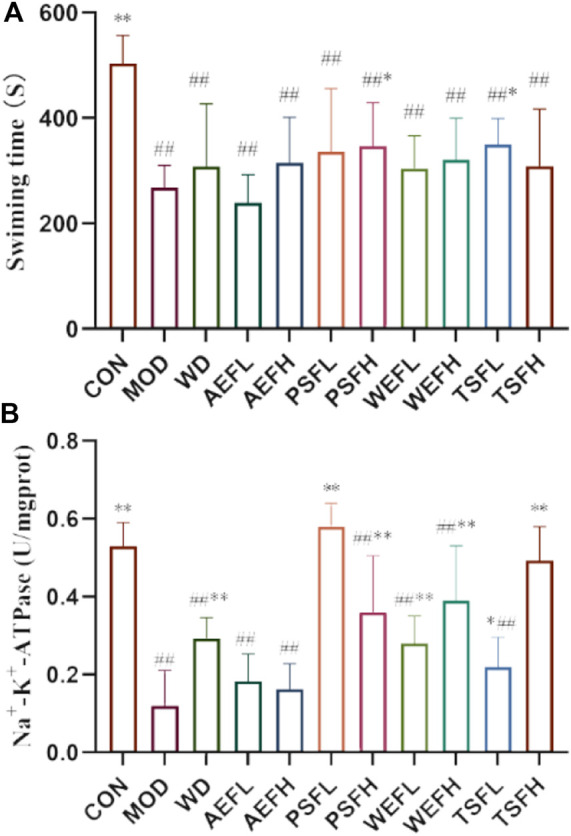
*qi*-tonifying effects of BG and its fractions. **(A)** Effects of BG and its fractions on weight-loaded swimming time in rats. **(B)** Effects of BG and its fractions on Na^+^–K^+^-ATPase.

The mean swimming time until exhaustion in the MOD group was significantly reduced compared with that in the CON group, as shown in Figure 1. Exercise endurance in weight-loaded swimming rats was enhanced following administration with PSFH and TSFL.

### 3.2 Echocardiographic assessment

**FIGURE 2:**
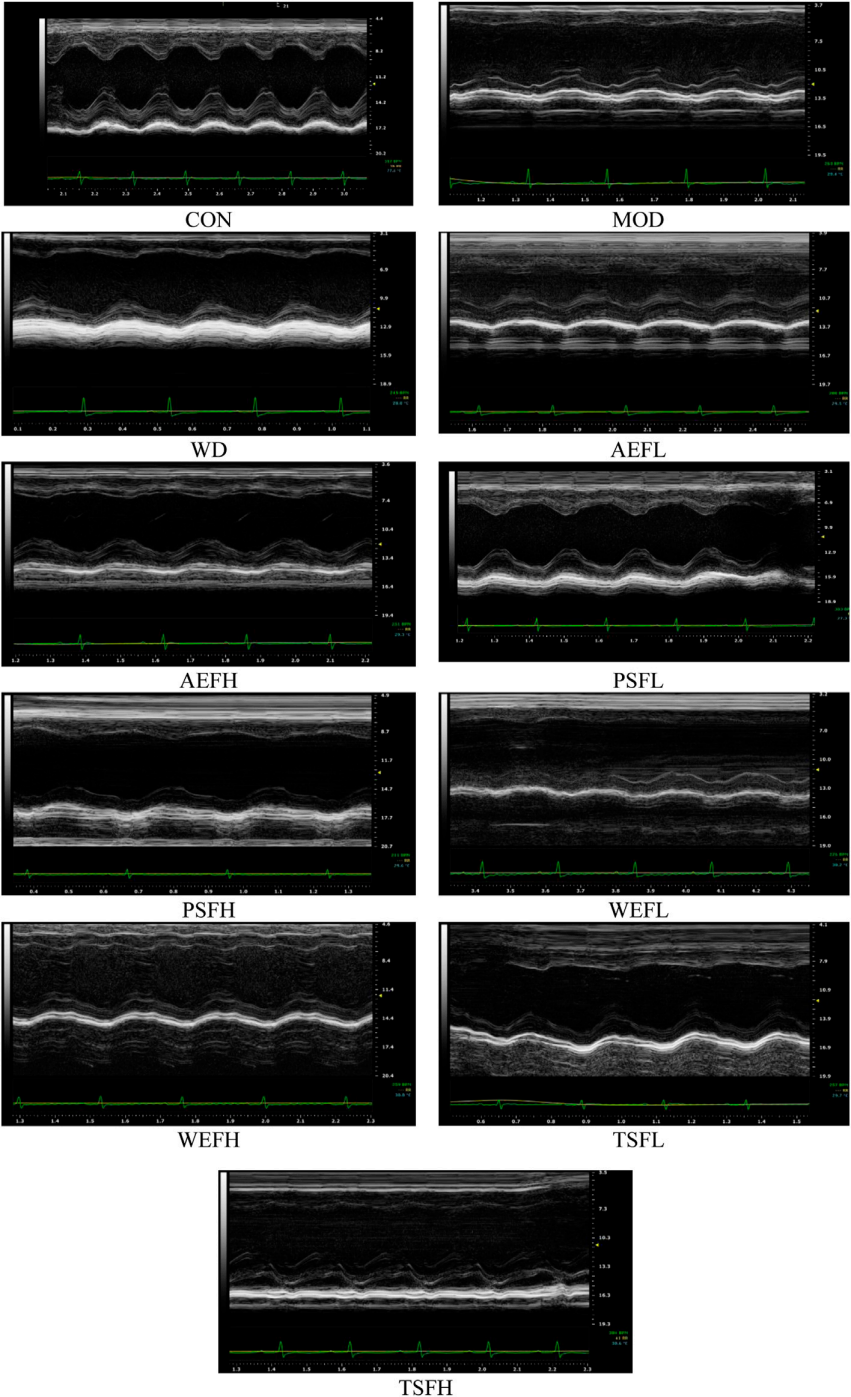
Effects of BG and its fractions on echocardiographic examinations.

The echocardiography parameters for assessing the structure and function of the left ventricle as shown in Supplementary Table S1. The MOD group demonstrated decreased EF and FS values when compared to the CON group, while the LVID s and LVID d values increased in comparison to the values in the CON group. Following a 2-week administration of BG and its fractions at varying doses to animals, the observed figures in Figure 2 demonstrated an increase in EF and FS.

### 3.3 Effects of BG and its fractions on histological evaluation

**FIGURE 3:**
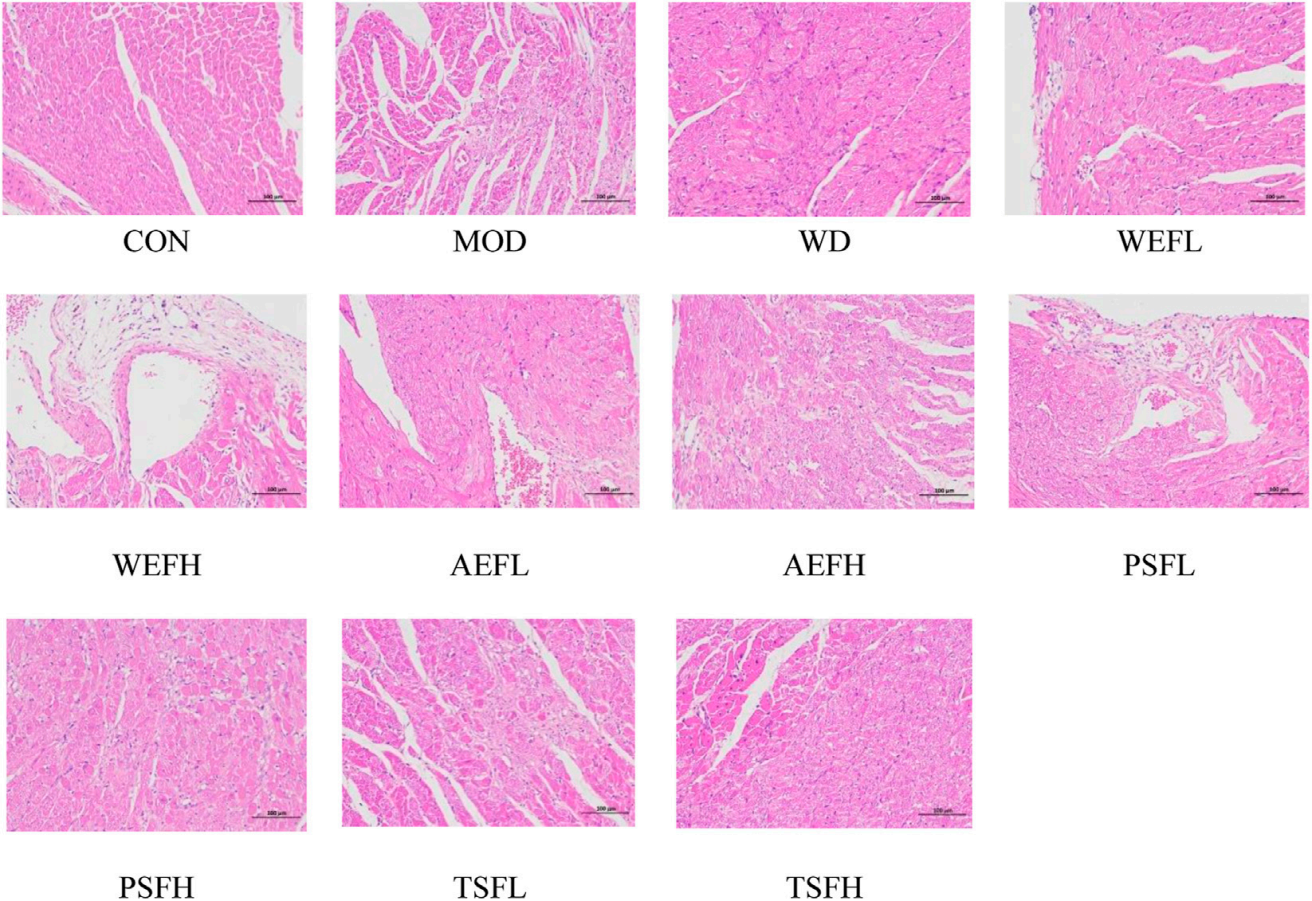
Effects of BG and its fractions on HE staining.

**FIGURE 4:**
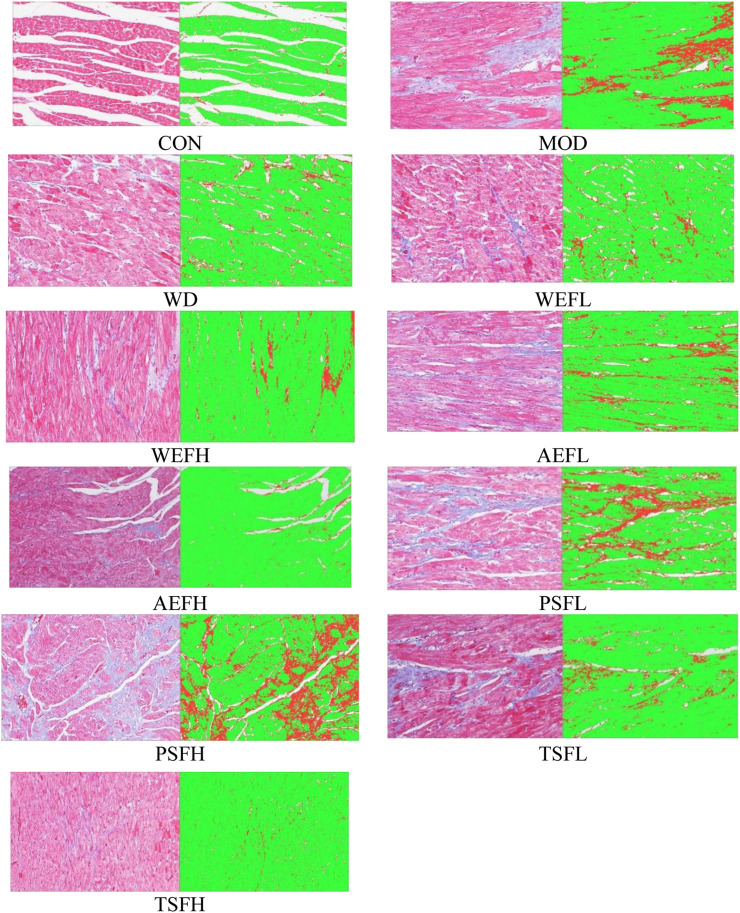
Effects of BG and its fractions on Masson’s trichrome staining.

Figure 3 shows that the CON group had normal characteristics, with an ordered arrangement of myocardial cells. In contrast, myocardial cells in the MOD group showed significant enlargement, hypertrophy, and disorganized arrangement, accompanied by an increase in intercellular substances. However, in the treatment groups of BG and its fractions, these pathological phenotypes were largely replaced by well-arranged myocardial cells. Additionally, Masson’s trichrome staining showed that red myocardial cells and blue collagen were clearly visible. Based on the staining results, it was observed that rats in the CON group had no discernible collagen deposition in their myocardial tissue. In contrast, rats in the MOD group displayed a substantial presence of blue collagen within their myocardial cells, with a significantly higher collagen volume fraction compared to that of the CON group. Treatment with WD, AEFL, AEFH, WEFH, TSFL, and TSFH resulted in varying degrees of restoration of collagen deposition within the myocardial cells, with the CVF being lower than that of the MOD group, as depicted in Figure 4 and Supplementary Figure S5.

### 3.4 Changes in pro-inflammatory cytokines and myocardial enzymes

**FIGURE 5:**
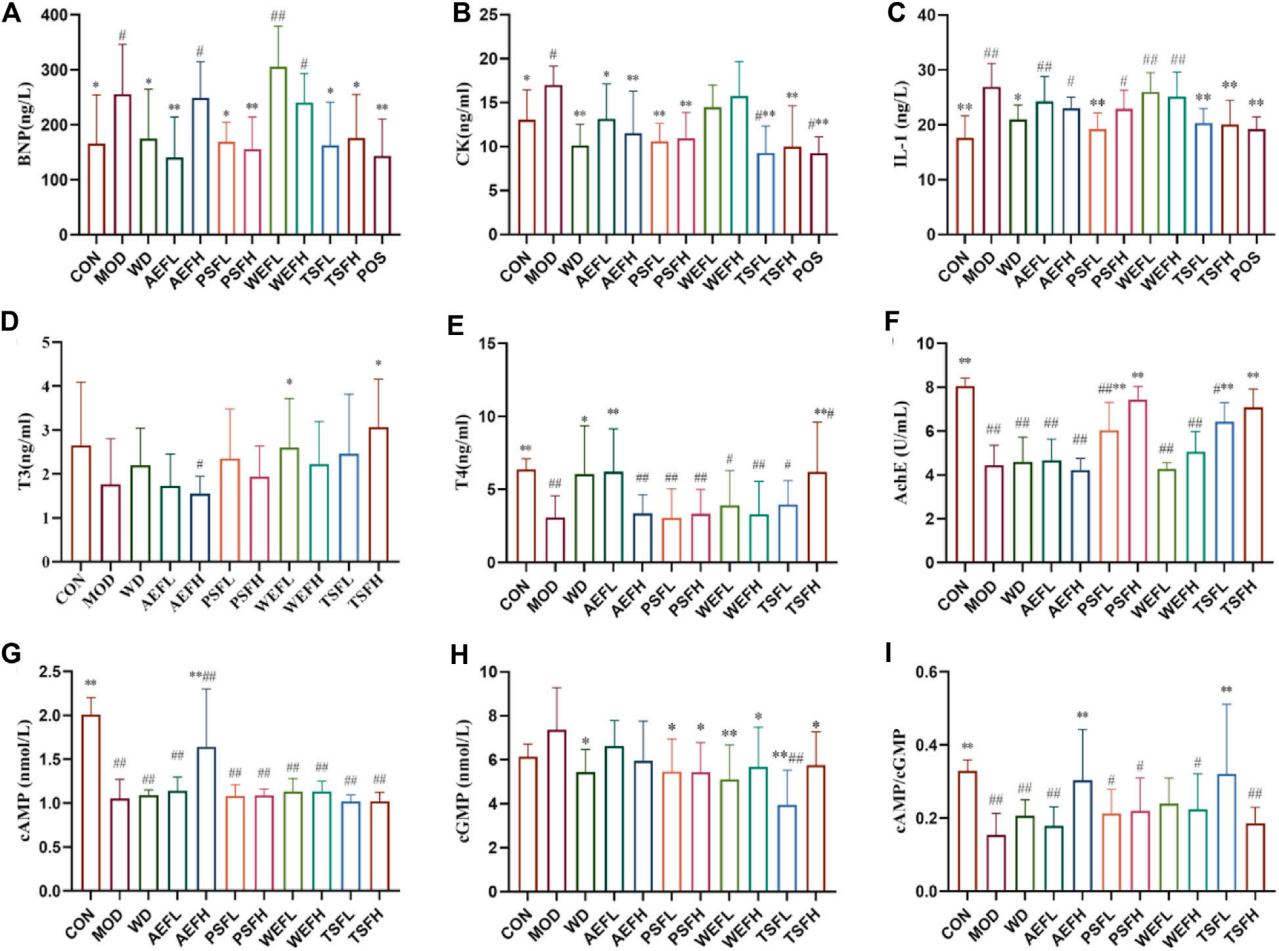
Effects of BG and its fractions on anti-HF action. Rats' cumulative values are represented as means ± standard deviations, with n = 8. Using the ANOVA analysis with LSD, the values were *p < 0.05 and **p < 0.01 in comparison with the MOD group and ^#^p < 0.05 and ^##^p < 0.01 in comparison with the CON group.

Figures 5A–C show that the levels of BNP, CK, and IL-1 in the MOD rats are significantly higher than those in the CON group. The treatments of WD, AEFL, PSFL, PSFH, TSFL, TSFH, and POS significantly inhibited the upregulation of BNP and CK expressions induced by HF compared to that of the MOD group. In addition, AEFH can also reduce the increased content of CK caused by HF. The levels of IL-1 in WD, PSFL, TSFL, TSFH, and POS decreased compared with those in the MOD group.

### 3.5 Effects of BG and its fractions on thyroid hormones

As shown in Figures 5D, E, the levels of T3 and T4 were lower in the MOD group than in the CON group, and the levels of T4 were statistically different. The levels of T3 and T4 were significantly increased by the administration of TSFH compared with the levels in the MOD group. The concentration of T3 in the WEFL group increased compared with that in the MOD group. The increased levels of T4 in the WD and AEFL groups, compared with those in the MOD group, were statistically significant.

### 3.6 Effects of BG and its fractions on AChE and Na^+^–K^+^-ATPase

The result revealed that the activity of AChE and Na^+^–K^+^-ATPase in MOD rats decreased when compared to that in CON rats. Treatment with PSFL, PSFH, TSFL, and TSFH activated the activity of AChE, and treatment with WD, PSFL, PSFH, WEFL, WEFH, TSFL, and TSFH activated the activity of Na^+^–K^+^-ATPase when compared to the MDL rats, as presented in Figures 5F, 1B.

### 3.7 Effects of BG and its fractions on cAMP and cGMP

As shown in Figures 5G–I, compared with the CON groups, the ratio of cAMP/cGMP in the serum of MOD rats decreased significantly, suggesting that Yang-deficiency syndrome occurred in those rats. Compared with the MOD group, the AEFH and TSFL groups showed an increase in the cAMP/cGMP ratio in the serum.

### 3.8 Targeted validation of the steroids and effects of BG and its fractions on progestogens

**FIGURE 6:**
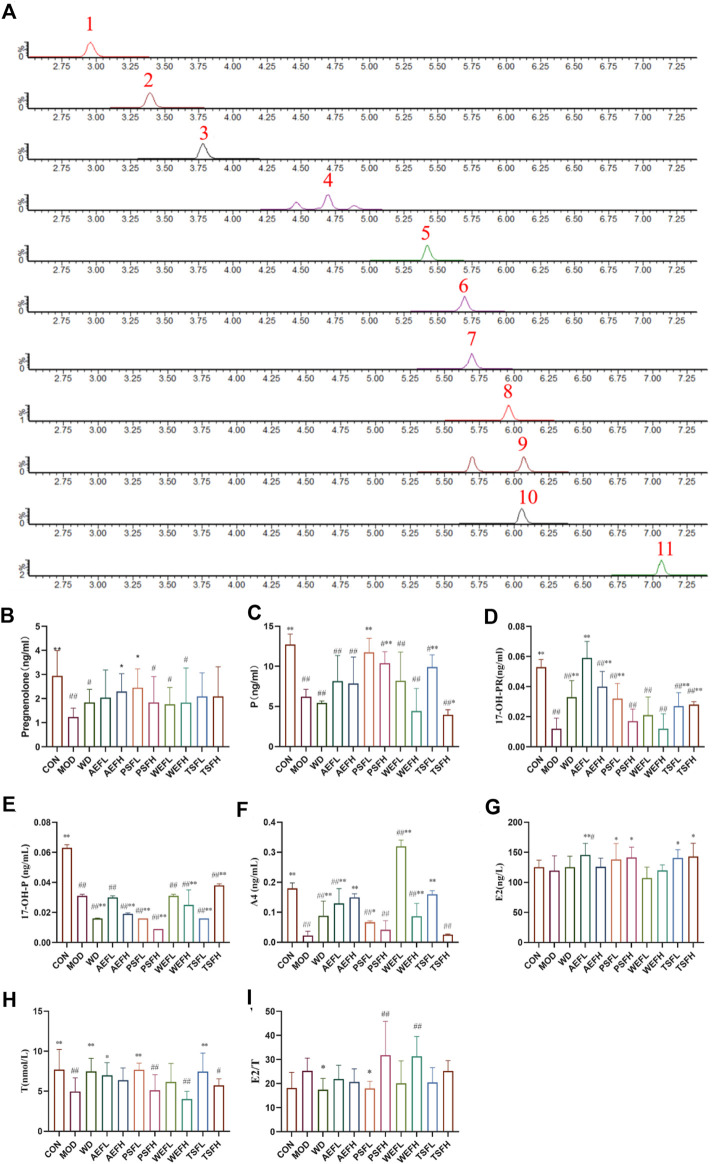
Chromatographic separation of steroids in serum samples (MRM) and effects of BG and its fractions on progestogens. Note: 1: aldosterone; 2: cortisone; 3: cortisol; 4: 21-deoxycortisol; 5: corticosterone; 6: androstenedione; 7: DHEA (dehydroepiandrosterone); 8: 17a-hydroxyprogesterone; 9: 17-OH-pregnenolone; 10: progesterone; 11: pregnenolone.

Under the ultra-performance liquid chromatography–triple quadrupole tandem mass spectrometry (UPLC-QqQ-MS/MS) conditions, the chromatographic separation of the steroids was achieved within 7 min, as shown in Figure 6A. Quantitative analysis was performed using MRM modes for nine analytes in the positive ion mode, including A4, 17-OH-P, P, pregnenolone, DHEA, COR, CORT, cortisone, and 21-OH-P, and for the two analytes ALDO and 17-OH-PR, as shown in Table 3. As depicted in Figures 6B–E, pregnenolone, P, 17-OH-PR, and 17-OH-P were significantly decreased in the MOD group compared to those in the CNH group. The AEFH and PSFL groups upregulated the levels of pregnenolone compared with the MOD group. The levels of P were significantly increased in the PSFL, PSFH, TSFL, and TSFH groups compared with those in the MOD group. The content of 17-OH-PR in the AEFL, AEFH, PSFL, TSFL, and TSFH groups significantly increased. The content of 17-OH-P in the WD, AEFH, PSFL, PSFH, WEFH, TSFL, and TSFH groups significantly decreased.

**TABLE 3:** Analytical parameters of an 11-steroid plasma panel using LC–MS/MS.

Compound	Formula	Parent ion (m/z)	Daughter Ion (m/z)	Collision energy (/ev)	Cone voltage (v)	Retention time (min)
Aldosterone	C_21_H2_8_O_5_	359.23	189.11	18	20	2.92
			311.10	16	20	
Cortisone	C_21_H_28_O_5_	361.23	121.04	32	50	3.35
			163.10	12	50	
Cortisol	C_21_H_30_O_5_	363.30	121.08	8	50	3.75
			327.19	7	50	
21-Deoxycortisol	C_21_H_30_O_4_	347.27	311.23	14	50	4.40
			121.08	22	50	
Corticosterone	C_21_H_30_O_4_	347.30	91.05	50	50	4.61
			121.08	18	50	
Androstenedione	C_19_H_26_0_2_	287.30	97.03	10	50	5.42
			109.06	15	50	
DHEA (dehydroepiandrosterone)	C_19_H_28_O_2_	271.23	197.20	18	40	5.91
			213.20	15	40	
17a-Hydroxyprogesterone	C_21_H_30_O_3_	331.30	97.04	25	40	6.02
			109.06	14	40	
17-OH-Pregnenolone	C_21_H_32_O_3_	331.08	287.16	20	50	6.03
			303.22	18	50	
Progesterone	C_21_H_30_O_2_	315.30	97.06	7	50	6.75
			109.02	10	50	
Pregnenolone	C_21_H_32_O_2_	299.10	159.11	20	30	7.03
			281.06	14	30	

### 3.9 Effects of BG and its fractions on androgens and estrogens

The serum levels of A4 and T in the MOD group decreased compared to those in the CON group. In comparison with the MOD group, the levels of A4 increased in the WD, AEFL, AEFH, PSFL, WEFL,WEFH, and TSFL groups. The levels of E2 in the AEFL, PSFL, PSFH, TSFL, and TSFH groups were significantly increased compared to those in the MOD group. For the E2/T ratio, compared to the MOD group, the ratio of E2/T in the WD and PSFL groups was decreased, as depicted in Figures 6F–I.

### 3.10 Effects of BG and its fractions on adrenaline hormones and ALDO

**FIGURE 7:**
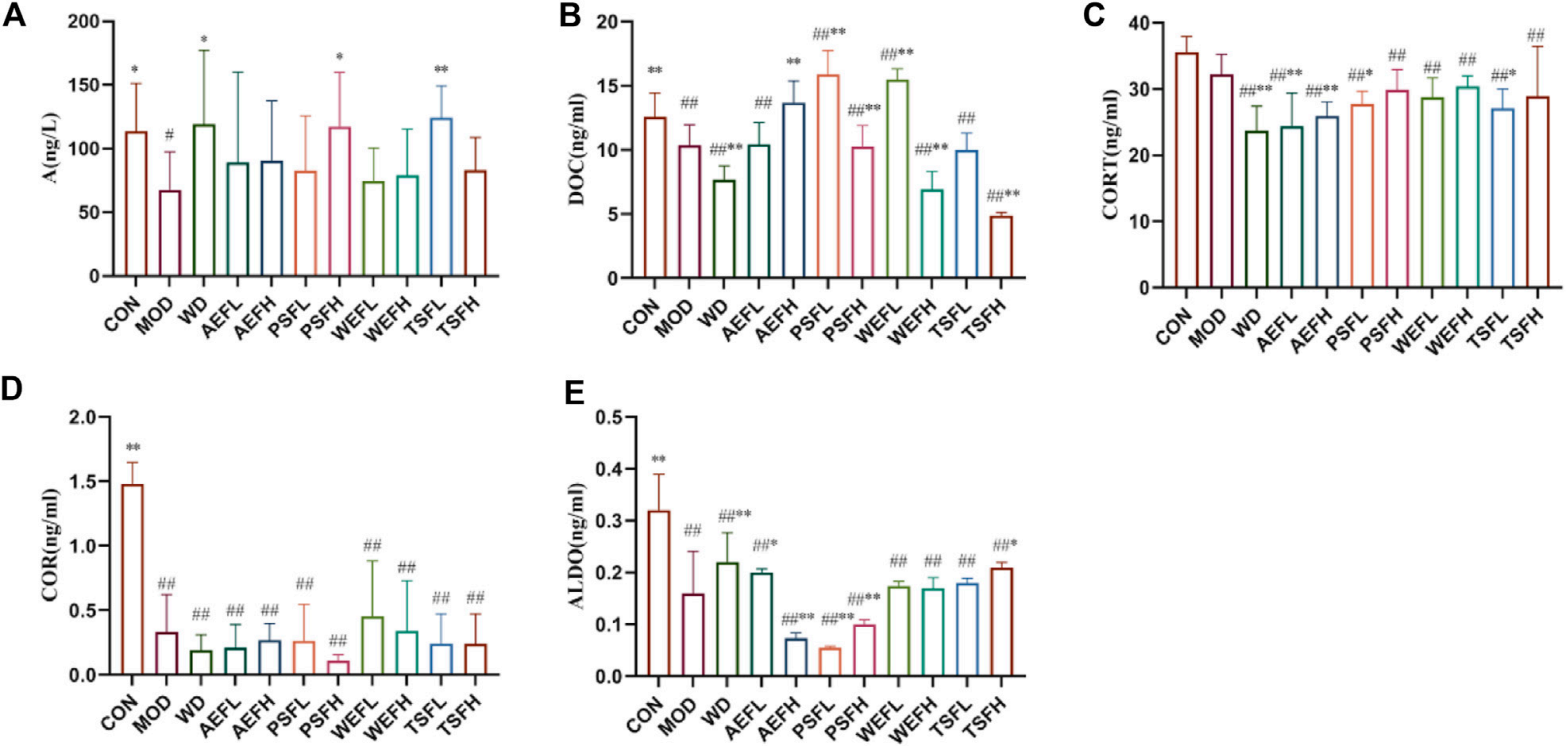
Effects of BG and its fractions on adrenaline hormones and ALDO. Rats' cumulative values are represented as means ± standard deviations, with n = 8. Using the ANOVA analysis with LSD, the values were ^*^p < 0.05 and ^**^p < 0.01 in comparison with the MOD group and ^#^p < 0.05 and ^##^p < 0.01 in comparison with the CON group.

Compared with that in the CON group, the contents of A, DOC, CORT, COR, and ALDO in the MOD group were all decreased. The contents of A, DOC, and ALDO all increased in the WD group compared with those in the MOD groups. The AEFH, PSFL, PSFH, WEFL, WEFH, and TSFH groups significantly increased the contents of DOC; meanwhile, D, AEFL, AEFH, PSFL, and TSFL significantly decreased the contents of CORT compared with that in the MOD group. The contents of ALDO in the AEFL, PSFH, and TSFH groups all increased, and the contents of ALDO in the AEFH and PSFH decreased, as shown in Figure 7A–E.

### 3.11 Effects on the intestinal flora

**FIGURE 8:**
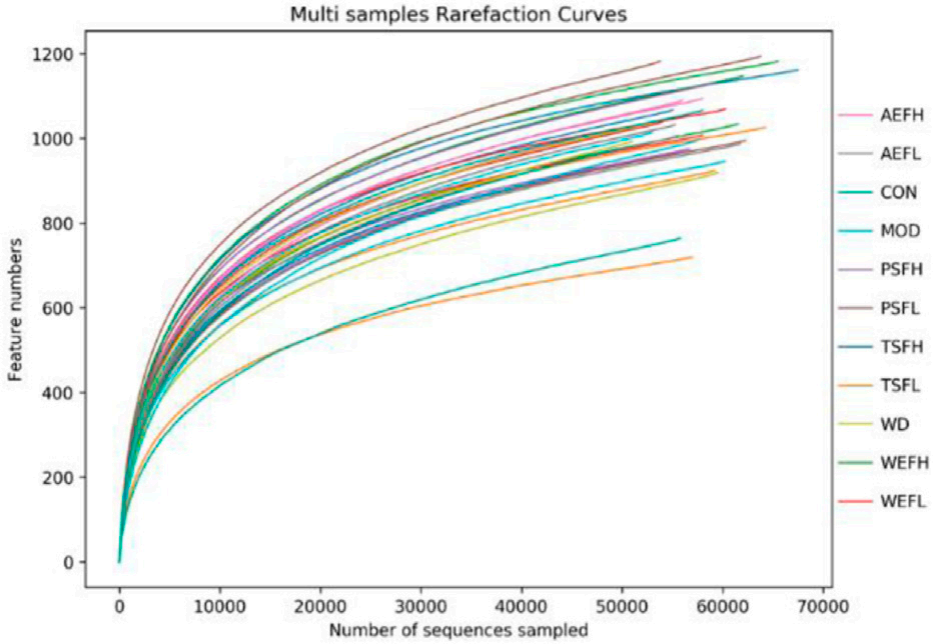
Rarefaction curves.

**FIGURE 9:**
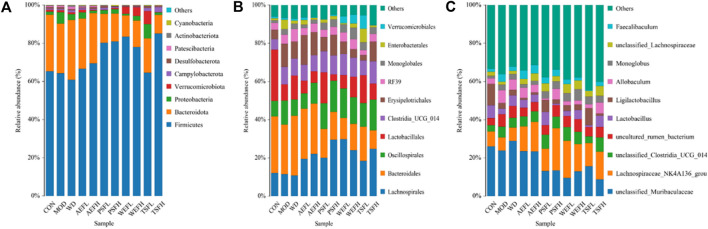
Distribution of intestinal flora at the phylum level. **(A)** Distribution of intestinal flora at the phylum level. **(B)** Distribution of intestinal flora at the order level. **(C)** Distribution of intestinal flora at the genus level.

**FIGURE 10:**
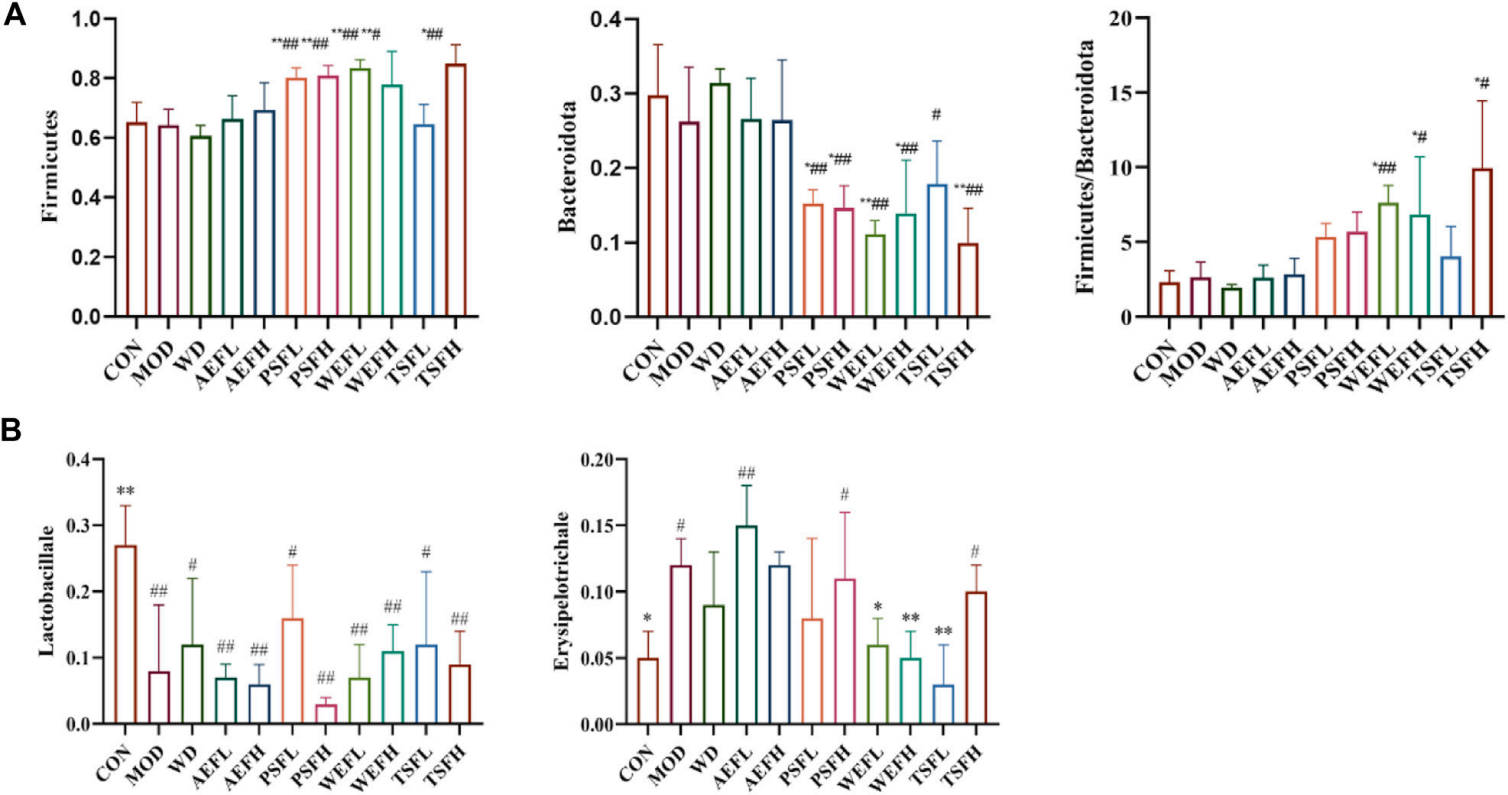
Level of gut microbiota in phylum levels and order levels. **(A)** The levels of *Firmicutes*, *Bacteroides,* and *Firmicutes*/*Bacteroides* in phylum levels. **(B)** Level of *Lactobacillales* and *Erysipelotrichales* in phylum levels. Rats' cumulative values are represented as means ± standard deviations, with n = 8. Using the ANOVA analysis with LSD, the values were *p < 0.05 and **p < 0.01 in comparison with the MOD group and ^#^p < 0.05 and ^##^p < 0.01 in comparison with the CON group.

Within a certain range, the sparse curve tended to be flat with the increase in the number of sequences sampled, as shown in Figure 8, indicating that the sequencing volume of each sample in this study was sufficient. As shown in Figure 9A–C, at the phylum level, the gut microbiota of each group of rats is composed of *Firmicutes*, *Bacteroides*, *Proteobacteria*, *Verrucomicrobia*, *Campylobacterota*, *Desulfobacterota*, *Patescibacteria*, *Actinobacteria*, and *Cyanobacteria*, with *Firmicutes* and *Bacteroides* having the highest relative abundance. Although the types of gut microbiota in each group did not change, their relative abundance changed. As shown in Figure 10A, in comparison with the CON group, the proportion of *Firmicutes* and *Bacteroides* in the gut microbiota of the MOD group increased. *Lactobacillales* significantly decreased in the MOD group, while *Erysipelotrichales* significantly increased, as shown in Figure 10B. In comparison with that of the MOD group, the *Lactobacillales* in the gut microbiota of the WEFL, WEFH, and TSFL groups increased.

The rats’ cumulative values are presented as means ± standard deviations, with n = 8. Using the ANOVA analysis with LSD, the values were ^*^ *p* < 0.05 and ^**^ *p* < 0.01 in comparison with the MOD group and ^#^ *p* < 0.05 and ^##^ *p* < 0.01 in comparison with the CON group.

### 3.12 Effects on SCFAs

**FIGURE 11:**
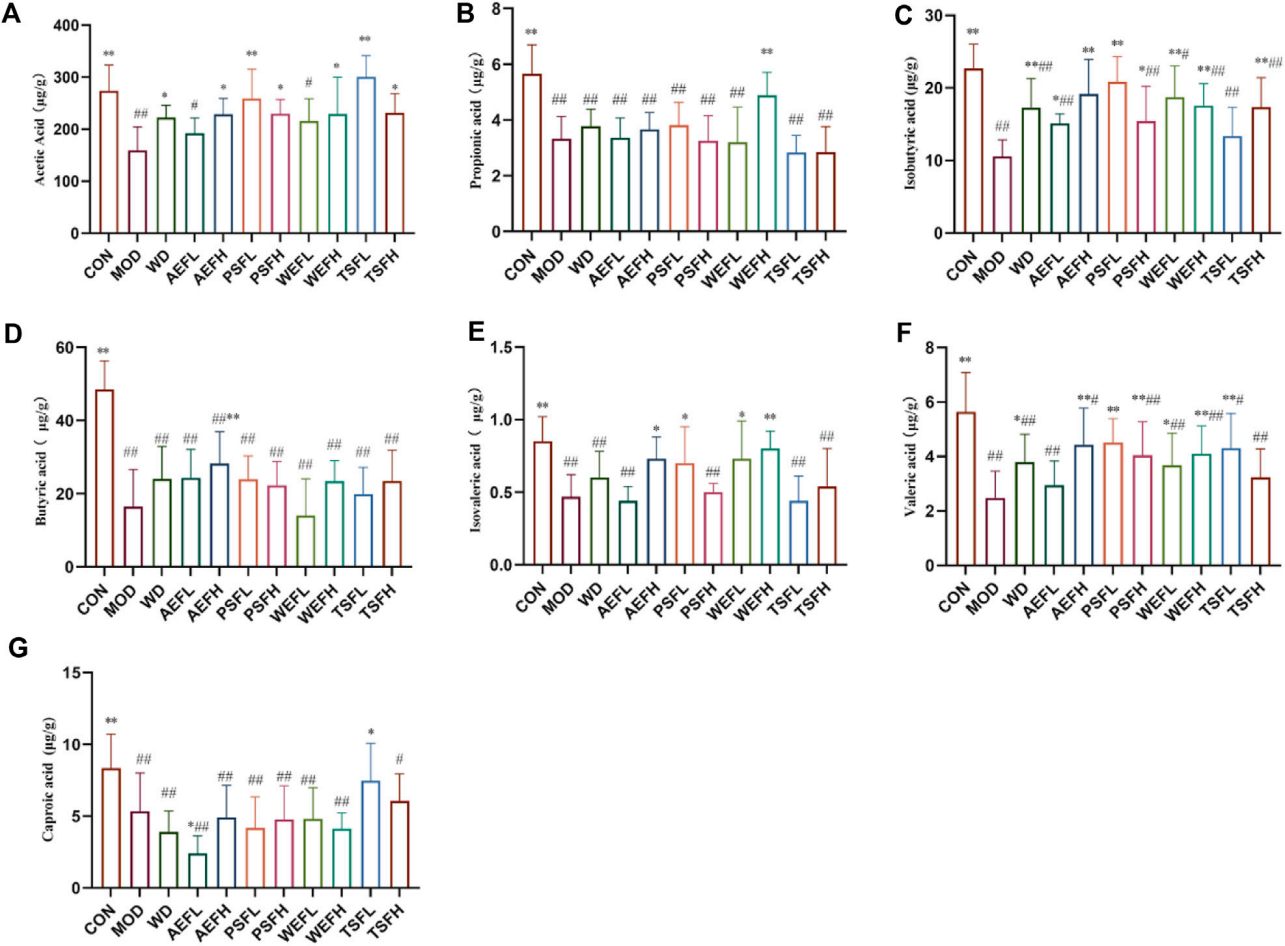
Content of SCFAs in the excrement of rats. Rats' cumulative values are represented as means ± standard deviations, with n = 8. Using the ANOVA analysis with LSD, the values were *p < 0.05 and **p < 0.01 in comparison with the MOD group and ^#^p < 0.05 and ^##^p < 0.01 in comparison with the CON group.

As shown in Figure 11A–G, in comparison with that in the CON group, the content of acetic acid, propionic acid, isobutyric acid, butyric acid, isovaleric acid, valeric acid, and caproic acid in the MOD group significantly reduced. Compared with that in the MOD group, the content of acetic acid in the WD, AEFH, PSFH, WEFH, TSFL, and TSFH groups significantly increased. The content of propionic acid in the WEFH group significantly increased, and the content of isobutyric acid in the WD, AEFL, AEFH, PSFL, PSFH, WEFL, WEFH, and TSFH groups is higher than that in the MOD group. In comparison with that in the MOD group, the content of butyric acid in the AEFH group significantly increased. Compared with that in the MOD group, the content of valeric acid in the WD, AEFH, PSFL, WEFL, and WEFH groups significantly increased. The content of isovaleric acid in the AEFH, PSFL, WEFL, and WEFH groups significantly increased, and the content of caproic acid in the AEFL and TSFL groups was higher than that in the MOD group. The BPC chromatograms of each group are depicted in Supplementary Figures S5–S16.

### 3.13 Protective effect of ginsenosides on ISO-induced H9C2 cells

**FIGURE 12:**
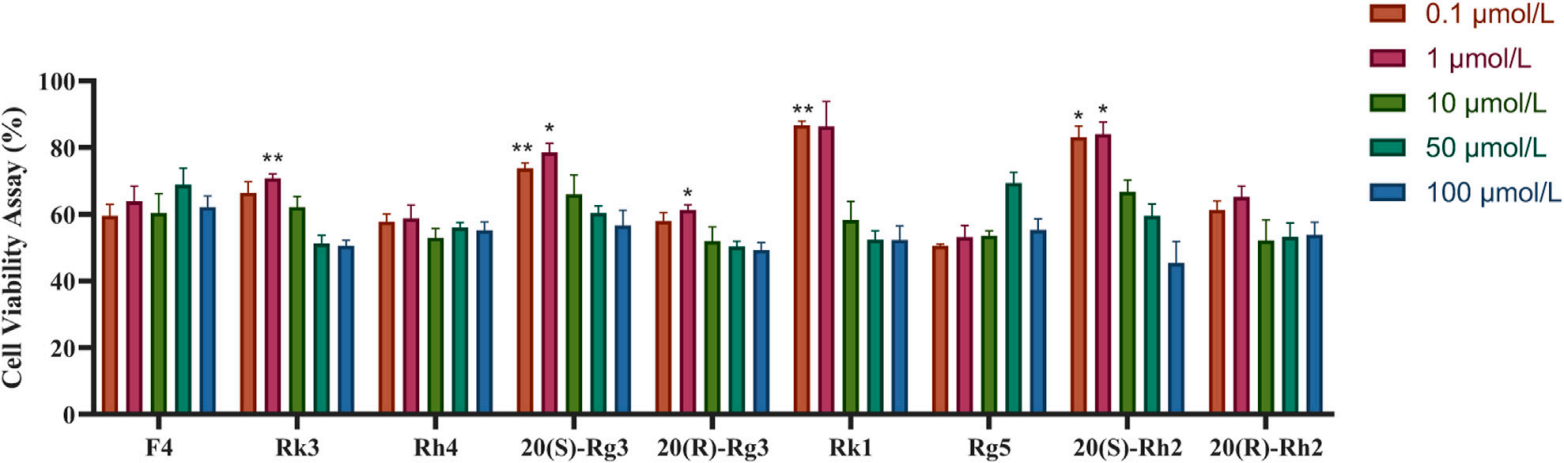
Protective effect of ginsenosides on ISO-induced H9C2 cells.

Ginsenosides 20-(S)-Rg3, Rk1, and 20-(S)-Rh2 have the strongest protective effect when the concentration is 0.1 μmol/L, and when the concentration is 1 μmol/L, Rk3, 20-(S)-Rg3, and 20-(S)-Rh2 have the strongest protective effect, as shown in Figure 12.

### 3.14 PCA of the biochemical indexes

**FIGURE 13:**
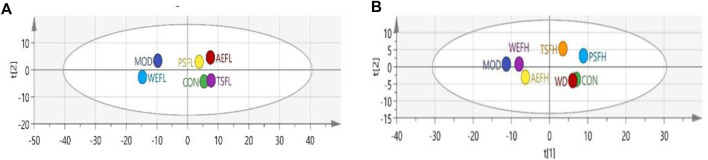
Analysis of PCA score plots. **(A)** Analysis of PCA score plots for all indices *in vivo* of the CON, MOD, WEFL, TSFL, AEFL, and PSFL groups. **(B)** Analysis of PCA score plots for all indices *in vivo* of the CON, MOD, WD, WEFH, TSFH, AEFH, and PSFH groups.

The results showed that the MOD and CON groups were well separated, and after treatment, TSFL, AEFL, and PSFL showed values closer to those of CON, as shown in Figure 13A. The WD group showed obvious overlap with the CON group. Furthermore, PSFH and TSFH had an obvious tendency toward the CON group, as shown in Figure 13B.

## 4 Discussion

In TCM, ginseng is classified as a *qi*-tonifying medicine and is especially regarded as having the action of reinforcing vital energy (*Yuan qi*). Clinical experience indicated that ginseng was mostly applied to diseases caused by shock, acute heart failure, and hemorrhage. Nowadays, it is mainly used for chronic diseases that last for a longer time, such as chronic HF. Our previous research revealed that the *qi*-tonifying effect of ginseng is associated with the HPT and HPA axes and energy metabolism marked by mitochondrial Na^+^–K^+^-ATPase activity, all of which are associated with energy metabolism and substance metabolism in the organism. This fact is consistent with the modern realization that the function of *qi* is mainly involved in the energy modulation of organisms. In addition, TCM believes that “the heart and the small intestine share a paired relationship,” visualizing the interaction between the heart and the small intestine, and research has also revealed that the intestinal flora participates in recovery from HF.

In our experiment, the analysis of echocardiogram and histology indicated that the HF model was established. Furthermore, the WD, AEF, PSF, and TSF groups showed anti-HF actions based on the levels of BNP and CK. The comparison of the swimming time of the drug administered and the MOD groups revealed that the PSFH and TSFL groups showed an obvious increase, indicating their stronger *q*i-tonifying effect. BNP is a sensitive biomarker of HF, and research suggests that the ratio of CORT/DHEAS in the plasma is significantly correlated with plasma BNP levels. The changes in cortisol and HF levels may be related to the activation of the PI3K/Akt signaling pathway following the activation of the glucocorticoid receptor. The PI3K/Akt signaling pathway is an important regulator of cell proliferation, cell cycle progression, and cell survival. Activated PI3K/Akt signaling leads to the phosphorylation of eNOS, increasing the production of nitric oxide (NO) (Moriyama et al., 2000).

The thyroid is the largest endocrine organ in the body, and synchronizing thyroid hormone and regulating metabolism are its main functions. Under normal physiological conditions, hypothalamic neurons secrete (thyrotropin-releasing hormone (TRH), which promotes the secretion of thyroid-stimulating hormone (TSH) by pituitary neurons in the HPT axis. Through its interaction with the thyroid, TSH regulates T3 and T4 in the blood, and T3 and T4 control TRH and TSH via feedback, forming an HPT axis regulation loop. In this experiment, the T3 and T4 levels were lower in the MOD group compared with those in the CON group, and T4 levels were statistically different. The levels of T3 and T4 were significantly increased by the administration in the TSFH group compared with the levels in the MOD group. The concentration of T3 in the WEFL group increased compared with that in the MOD group. The increased levels of T4 in the WD and AEFL groups compared with those in the MOD group were statistically significant. Thyroid hormone stimulates the activation of the HPA axis through hypothalamic secretion of the CRF, which stimulates the pituitary gland to secrete the adrenocorticotropic hormone ACTH. This, in turn, causes COR to be released by the adrenal glands. In this experiment, compared to the CON group, the MOD group showed significant decreases in CORT, COR, and DHEA. Compared to the MOD group, the WD AEFH, PSFL, TSFL, and TSFH groups showed significant increases. The WD, AEFH, PSFL, TSFL, and TSFH groups showed significant increases in DHEA. Reactive oxygen species (ROS) and reactive nitrogen species (RNS) are products of cellular metabolism and are directly associated with all of the important characteristics of cardiovascular disease—pathology and physiology. ROS can be produced by complexes such as NAD(P)H-oxidase or Nox in the cell membrane, by organelles, and by substances in the cytoplasm. Additionally, low levels of the limiting cofactor and substrate tetrahydrobiopterin and l-arginine can lead to the uncoupling of eNOS, resulting in decreased production of NO and increased production of ROS. Androgens such as T can directly stimulate pro-inflammatory enzymes (thromboxane synthase) in the vascular system, as well as cyclooxygenase-1 (COX-1) and COX-2 in the rat aorta and mesenteric artery. This may affect the production of ROS in vascular smooth muscle cells. In this experiment, black ginseng water decoction and its parts showed strong effects on androgens (Yu et al., 2014).

Research conducted previously indicated that cAMP and cGMP, the second messengers, played opposite actions in HF status, and cAMP/cGMP could be an indicator to reflect HF severity (Miller et al., 2011; Wang et al., 2012). Our results indicated that, compared with the CON groups, the ratio of cAMP/cGMP in the serum of the MOD rats decreased significantly. Compared with the MOD group, the AEFH and TSFL groups could increase the ratio of cAMP/cGMP in the serum, indicating the anti-HF action of the AEFH and TSFL groups.

Previous research has shown that various qualities of the ginseng medicinal herb can enhance the levels of adrenaline–cortisol (CORT, COR). *Warm*-nature ginseng medicines can significantly increase 17-OH-PR and P levels while simultaneously reducing 17-OH-P levels. In contrast, *cold*-nature ginseng medicines reduced A4 and T levels. *Warm*-nature ginseng medicines also increased the E2/T ratio and Na^+^–K^+^-ATPase activity, while *cold*-nature ginseng medicines had the opposite effect. Based on these findings, WD, AEF, PSF, and TSF tend to have a *warm* nature, and this may also be the reason why they have a stronger anti-HF effect. Interestingly, in this experiment, E2/T increased in the MOD group, while E2/T decreased in the WD and PSFL groups, suggesting that E2/T is related to HF and can be used as an indicator to evaluate HF.

Recent research indicated that improving vagal activity is one of the therapeutic ways to treat heart failure. When HF occurs, acetylcholine is released more from the postganglionic nerve fibers of the cardiac vagus nerve in HF to activate the AChE and acetylcholine M receptors, mainly the M2 receptor in the heart, which could regulate the expression of inflammatory factors, thereby protecting cardiomyocytes (Cheng et al., 2021; Zhang et al., 2021). In this experiment, the activity of AChE in the MOD group decreased when compared to that in the CON group. Treatment with PSFL, PSFH, TSFL, and TSFH activated the activity of AChE. Correspondingly, the level of IL-1 in WD, PSFL, TSFL, and TSFH decreased compared with that in the MOD group. Thus, AChE and IL-1 may reflect the effects of BG on the inflammation of HF through the vagus nerve.

As SCFAs are the products of gut microbiota fermenting dietary fiber, approximately 90%–95% of SCFAs are composed of acetate, propionate, and butyrate. Acetate can reduce the production of IL-6 and IL-8, while butyrate exerts anti-inflammatory effects by activating G protein-coupled receptor 43 and inducing the proliferation of regulatory T cells, thereby inhibiting the generation of Th17 cells and suppressing inflammatory responses and the progression of heart failure. In addition, propionate can also improve cardiac immune cell infiltration. The gut microbiota and their metabolites, including SCFAs, have been shown to regulate the abundance of *Firmicutes* and *Bacteroidetes*, which are associated with energy metabolism and may alleviate HF. Additionally, the TSFL group may regulate *Lactobacillus*/*Erysipelotrichales* to exert an anti-inflammatory effect. The main active components may be Rk1, Rk3, 20-(S)-Rg3, 20-(S)-Rh2. *Lactobacillus* has been shown to inhibit inflammation and oxidative stress and improve multiple organ ischemia-reperfusion injury (Yong et al., 2019). PSFH and TSFL may affect inflammatory cytokines through the gut microbiota (*Lactobacillus*/*Erysipelotrichales*) and their metabolites (acetate, butyrate) to exert an anti-inflammatory effect. These various mechanisms of action among the different groups reflect the significance and necessity of traditional Chinese medicine’s overall effect.

## 5 Conclusion

To sum up, our research could be summarized as follows:

1) The BG and all its split fractions demonstrated varying levels of efficacy in alleviating HF. TSF, primarily containing secondary ginsenosides, and PSF, consisting of polysaccharides, were identified as the active compounds responsible for BG’s efficacy in treating heart failure. The principal active components may include Rk1, Rk3, 20-(S)-Rg3, and 20-(S)-Rh2.
2) The decoction of BG and its components exhibited an increasingly potent impact on decreasing androgen hormones induced by HF. This phenomenon may be attributed to the activation of the eNOS-NO pathway through androgen regulation, thereby contributing to its anti-heart failure mechanism.
3) The WD, PSFH, and TSFL groups may exert anti-inflammatory effects through the intestinal flora (*Lactobacillaceae*/*Erysipelotrichaceae*) and its metabolites (acetic acid and butyric acid) and through the vagus nerve to act on the inflammatory factors (IL-1).
4) Our research first reported that E2/T is related to HF and can be used as an indicator to evaluate HF and that AChE and IL-1 could reflect the effects of BG on inflammation of HF through the vagus nerve.
5) Our research found that the first messenger E2/T and the second messenger cAMP/cGMP showed a similar change in HF, and their correlation mechanism requires further study to elucidate the regulation of androgen and estrogen on HF.

## Figures and Tables

**Table:** 

**AChE**	Acetylcholinesterase
**AD**	Adrenaline
**ALDO**	Aldosterone
**AEF**	Alcohol-eluted fraction
**A4**	Androstenedione
**BG**	Black ginseng
**BNP**	Brain natriuretic peptide
**CORT**	Corticosterone
**COR**	Cortisol
**CK**	Creatine kinase
**cAMP**	Cyclic adenosine monophosphate
**cGMP**	Cyclic guanosine monophosphate
**DHEA**	Dehydroepiandrosterone
**DOC**	Deoxycorticosterone
**ELISA**	Enzyme-linked immunosorbent assay
**E2**	Estradiol
**HPA**	Hypothalamus–pituitary–adrenal
**HPG**	Hypothalamic–pituitary–gonadal
**HPT**	Hypothalamus–pituitary–thyroid
**HF**	Heart failure
**HB**	Cortex Phellodendri
**IL-1**	Interleukin-1
**ISO**	Isoproterenol
**Na + -K + -ATPase**	Ultrafine Na^+^–K^+^-ATPase
**17-OH-P**	17-Hydroxyprogesterone
**17-OH-PR**	17-Hydroxypregnenolone
**P**	Progesterone
**PSF**	Polysaccharide fraction
**T**	Testosterone
**T4**	Thyroxine
**TCM**	Traditional Chinese medicine
**T3**	Triiodothyronine
**TSF**	Total saponin fraction
**UPLC-QqQ-MS/MS**	Ultra-performance liquid chromatography–triple quadrupole tandem mass spectrometry
**WD**	Water decoction
**WEF**	Water-eluted fraction

## Data Availability

The original contributions presented in the study are included in the article/Supplementary Material; further inquiries can be directed to the corresponding authors.
