# Supplementary material for: Black ginseng: a novel medicine for treating heart failure
Source: Front Pharmacol. 2024 Jul 18;15:1429214. doi: 10.3389/fphar.2024.1429214 (PMC11291204; doi:10.3389/fphar.2024.1429214)
Supplement: Supplementary file 1 [file DataSheet1.docx]

**Supplementary Material**

S1. Compounds of fractions in BG analyzed by HPLC


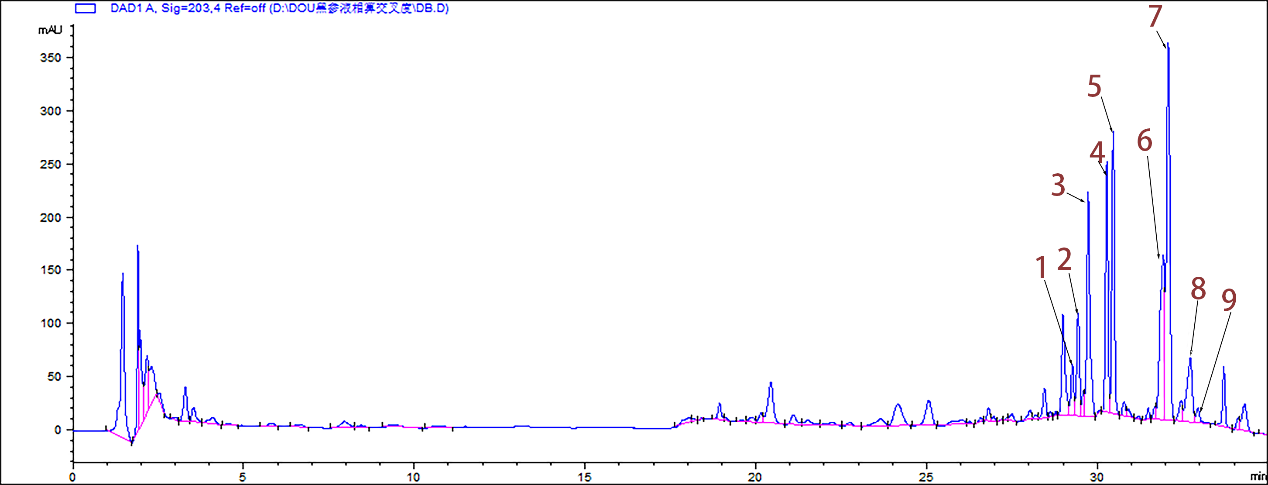


Figure S1 The main compounds of BG

Figure S2 The main compounds of WEF


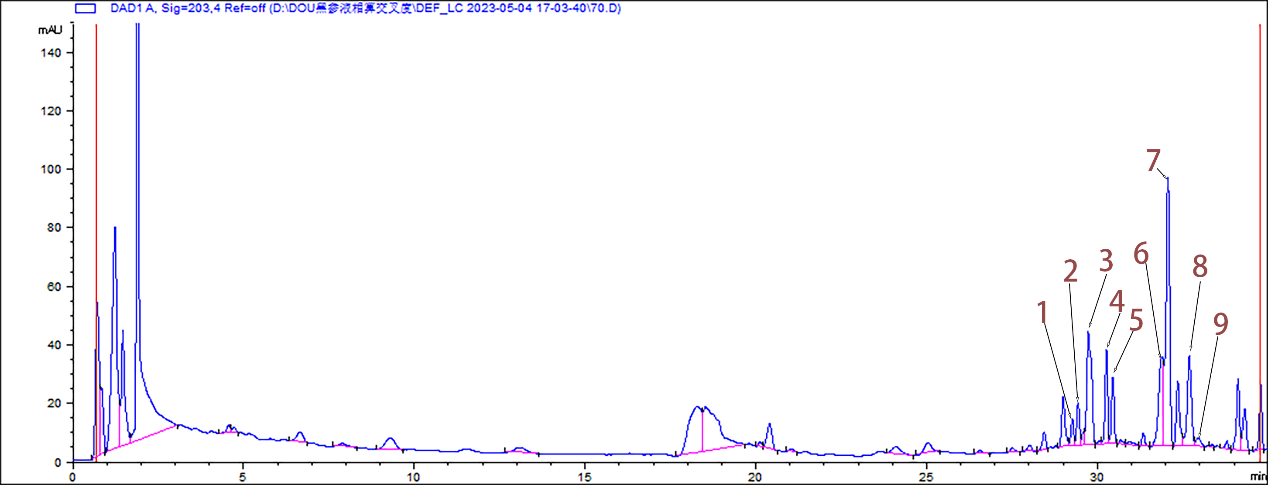


Figure S3 The main compounds of TSF


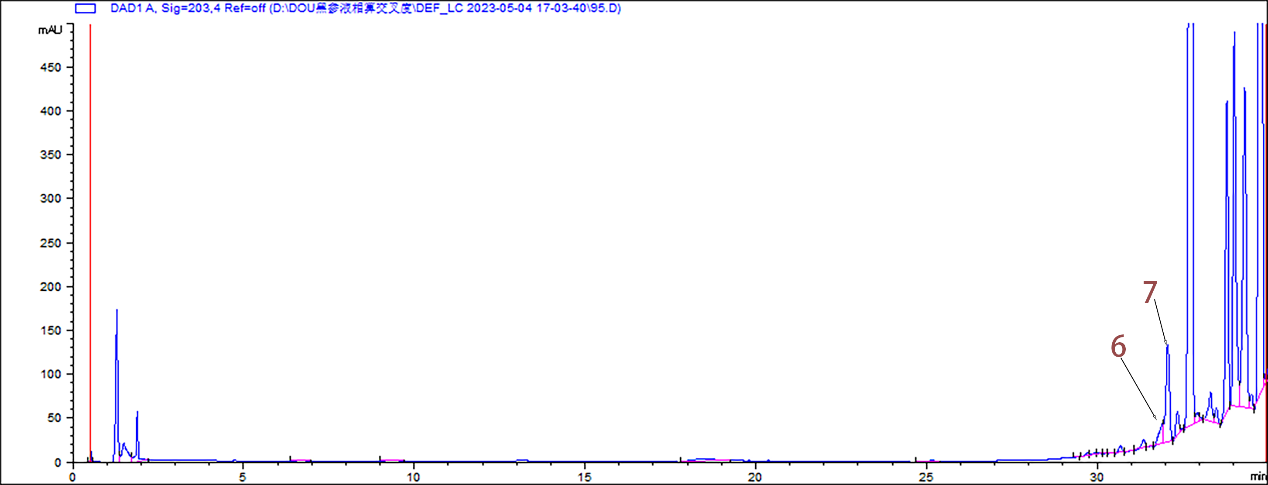


Figure S4 The main compounds of AEF

Note：1. F4；2. Rk3；3. Rh4； 4.20(S)-Rg3；5. 20-(R)- Rg3 ；6. Rk1；7. Rg5；8.20(R)-Rh ；9.20-(R)-Rh2

S2. Sequencing and analysis of intestinal flora

Total bacterial DNA was extracted from samples using the Power Soil DNA Isolation Kit (MO BIO Laboratories) according to the manufacturer’s protocol. DNA quality and quantity were assessed by the ratios of 260 nm/280 nm and 260 nm/230 nm. Then DNA was stored at −80◦C until further processing. The V3–V4 region of the bacterial 16S rDNA gene was amplified with the common primer pair (Forward primer, 5’-ACTCCTACGGGAGGCAGCA-3’; reverse primer,5’- GGAC TACHVGGGTWT

CTAAT-3’) combined with adapter sequences and barcode sequences. PCR amplification was performed in a total volume of 50μl, which contained 10μl buffer, 0.2μl Q5 high-fidelity DNA polymerase, 10μl high GC enhancer, 1μl dNTP, 10μM of each primer, and 60ng genome DNA. Thermal cycling conditions were as follows: initial denaturation at 95◦C for 5min, followed by 15 cycles at 95◦C for 1min, 50◦C for 1min, and 72◦C for 1min, with a final extension at 72◦C for 7min. The PCR products from the first step PCR were purified through VAHTSTM DNA clean beads. A second-round PCR was then performed in a 40μl reaction which contained 20μl 2 × Phusion HF MM, 8μl ddH_2_O, 10μM of each primer, and 10μl PCR products from the first step. Thermal cycling conditions were as follows: initial denaturation at 98◦C for the 30s, followed by 10 cycles at 98◦C for 10s, 65◦C for 30s min, and 72◦C for 30s, with a final extension at 72◦C for 5min. Finally, all PCR products were quantified by nanodrop 2000 and pooled together. High-throughput sequencing analysis of bacterial rDNA genes was performed on the purified, pooled sample using the Illumina Hiseq 2500 platform (2 × 250 paired ends) at Biomarker Technologies Corporation, Beijing, China

Table S1 Echocardiographic quantification evaluated the impaired cardiac function

| Groups | LVAW;d  （mm） | LVAW;s  （mm） | LVID;d  （mm） | LVID;s  （mm） | LVPW;d  （mm） | LVPW;s  （mm） | EF  （%） | FS  （%） | LV Mass AW  （mg） |
| --- | --- | --- | --- | --- | --- | --- | --- | --- | --- |
| CON1 | 1.45 | 2.60 | 8.02 | 3.78 | 1.79 | 2.94 | 82.36 | 52.89 | 769.32 |
| CON2 | 1.68 | 2.63 | 7.94 | 3.82 | 1.72 | 2.94 | 81.52 | 51.92 | 806.66 |
| CON3 | 1.58 | 2.44 | 7.16 | 3.79 | 1.74 | 2.95 | 77.05 | 47.02 | 662.15 |
| MOD1 | 1.05 | 1.13 | 6.86 | 5.51 | 1.15 | 1.43 | 39.40 | 19.72 | 353.70 |
| MOD2 | 1.27 | 1.35 | 5.60 | 4.77 | 1.48 | 1.78 | 31.03 | 14.81 | 340.87 |
| MOD3 | 1.32 | 1.52 | 6.73 | 5.34 | 1.26 | 1.54 | 41.07 | 20.65 | 422.62 |
| WD1 | 1.04 | 1.32 | 7.11 | 5.32 | 1.10 | 1.54 | 48.46 | 25.21 | 362.32 |
| WD2 | 1.21 | 1.32 | 7.03 | 5.47 | 1.24 | 1.56 | 43.54 | 22.18 | 425.58 |
| WD3 | 1.54 | 2.23 | 8.06 | 4.46 | 1.73 | 2.71 | 74.13 | 44.63 | 782.99 |
| AEFL1 | 1.24 | 1.28 | 5.14 | 3.96 | 1.65 | 2.08 | 45.83 | 22.99 | 322.14 |
| AEFL2 | 1.32 | 1.55 | 6.46 | 4.79 | 1.35 | 1.96 | 49.76 | 25.85 | 414.14 |
| AEFL3 | 1.65 | 1.74 | 8.06 | 5.47 | 1.44 | 1.99 | 58.37 | 32.07 | 724.66 |
| AEFH1 | 0.88 | 1.01 | 7.70 | 5.88 | 1.15 | 1.61 | 45.84 | 23.73 | 390.36 |
| AEFH2 | 1.21 | 1.28 | 7.05 | 5.52 | 1.21 | 1.76 | 42.60 | 21.63 | 418.53 |
| AEFH3 | 1.24 | 1.95 | 8.08 | 5.71 | 1.44 | 1.98 | 54.34 | 29.30 | 607.82 |
| PSFL1 | 1.38 | 1.93 | 8.25 | 5.64 | 1.56 | 2.18 | 57.64 | 31.61 | 707.24 |
| PSFL2 | 1.16 | 1.49 | 7.80 | 6.10 | 1.30 | 1.65 | 42.74 | 21.88 | 511.05 |
| PSFL3 | 1.43 | 1.65 | 7.57 | 5.12 | 1.57 | 2.35 | 58.98 | 32.37 | 629.24 |
| PSFH1 | 1.21 | 1.50 | 7.27 | 5.38 | 1.54 | 1.76 | 49.64 | 26.01 | 521.55 |
| PSFH2 | 1.21 | 1.39 | 7.36 | 5.57 | 1.34 | 1.83 | 46.95 | 24.34 | 483.90 |
| PSFH3 | 1.54 | 1.90 | 8.19 | 5.52 | 1.37 | 2.01 | 59.04 | 32.59 | 687.05 |
| WEFL1 | 1.07 | 1.44 | 6.58 | 5.04 | 1.55 | 1.73 | 45.95 | 23.52 | 416.57 |
| WEFL2 | 1.39 | 1.80 | 6.13 | 4.79 | 1.45 | 1.63 | 43.41 | 21.87 | 414.31 |
| WEFL3 | 1.39 | 1.72 | 8.01 | 5.51 | 1.54 | 1.84 | 57.15 | 31.20 | 668.79 |
| WEFH1 | 1.13 | 1.35 | 6.57 | 4.99 | 1.27 | 1.57 | 46.93 | 24.12 | 369.54 |
| WEFH2 | 0.99 | 1.23 | 7.35 | 5.74 | 1.05 | 1.56 | 42.93 | 21.89 | 362.18 |
| WEFH3 | 1.50 | 1.98 | 8.56 | 5.40 | 1.39 | 2.05 | 64.70 | 36.89 | 735.72 |
| TSFL1 | 1.54 | 1.60 | 7.14 | 5.17 | 1.34 | 1.76 | 52.24 | 27.65 | 540.63 |
| TSFL2 | 1.38 | 1.54 | 6.88 | 5.00 | 1.44 | 2.01 | 51.84 | 27.30 | 493.12 |
| TSFL3 | 1.50 | 2.27 | 7.42 | 3.99 | 2.05 | 2.94 | 76.19 | 46.31 | 767.99 |
| TSFH1 | 1.46 | 1.57 | 6.80 | 4.88 | 1.63 | 1.83 | 53.41 | 28.32 | 552.02 |
| TSFH2 | 1.16 | 1.35 | 7.40 | 5.30 | 1.05 | 1.66 | 53.18 | 28.34 | 405.50 |
| TSFH3 | 1.33 | 2.04 | 7.77 | 4.41 | 1.66 | 2.88 | 72.62 | 43.17 | 652.78 |

Figure S5 Effects of BG and its fractions on CVF

|  |
| --- |
| Figure S6 BPC chromatogram of CON |
|  |
| Figure S7 BPC chromatogram of MOD |
|  |
| Figure S8 BPC chromatogram of WD |
|  |
| Figure S9 BPC chromatogram of AEFL |
|  |
| Figure S10 BPC chromatogram of AEFH |
|  |
| Figure S11 BPC chromatogram of PSFL |
|  |
| Figure S12 BPC chromatogram of PSFH |
|  |
| Figure S13 BPC chromatogram of TSFL |
|  |
| Figure S14 BPC chromatogram of TSFH |
|  |
| Figure S15 BPC chromatogram of WEFL |
|  |
| Figure S16 BPC chromatogram of WEFH |
